# The Effects of Super-Fast Heating Rate and Holding Time on the Microstructure and Properties of DP Fe-0.16C-1.4Mn Sheet Steel

**DOI:** 10.3390/ma17204982

**Published:** 2024-10-11

**Authors:** Jiazheng Zhao, Jian Wang, Jun Li, Shengen Zhang, Fenghua Luo

**Affiliations:** 1 Institute for Advanced Materials and Technology, University of Science and Technology Beijing, Beijing 100083, China; b20200631@xs.ustb.edu.cn; 2 Research Institute, Baoshan Iron & Steel Co., Ltd., Shanghai 201999, China; wang-jian@baosteel.com (J.W.); lijun@baosteel.com (J.L.); 3 State Key Laboratory of Powder Metallurgy, Central South University, Changsha 410083, China

**Keywords:** super-fast heating, dual-phase sheet steel, mechanical properties, microstructure, austenite nucleation, heterogeneous structures

## Abstract

This study investigates the influence of super-fast heating rate and holding time on the microstructure and mechanical properties of dual-phase (DP) Fe-0.16C-1.4Mn sheet steel. Super-fast heating and cooling rates were achieved via induction heating and gas quenching. The results were also compared with those for a conventional low-speed heat treatment. The microstructures were characterized in detail using X-ray diffraction, scanning electron microscopy, electron backscatter diffraction, and electron probe microanalysis. The results showed that the layered structure of the DP Fe-0.16C-1.4Mn steel after super-fast heating was mainly composed of recrystallized ferrite, martensite clusters, and a small amount of residual austenite. Compared with the conventional method, super-fast heating significantly refined the grains and improved yield and tensile strength, but it slightly reduced the elongation. The fraction of martensite, which depends on the nucleation and growth behavior of austenite, was significantly affected by the heating rate and holding time. The DP structure of Fe-0.16C-1.4Mn steel had an atypical layered heterogeneous structure, with an uneven plastic strain between the two phases occurring during the deformation process, which is something that can improve fracture elongation.

## 1. Introduction

Steel is widely used as a fundamental structural material in the construction industry. As the steel industry develops, there is an increasing demand for high-strength and high-ductility structural steel [1,2,3]. In traditional production, sheet steel is slowly heated to a high temperature to homogenize its microstructure. If the steel also has a specific chemical composition, it can be cooled at a controlled rate to obtain the desired microstructure and mechanical properties [4]. The production of sheet steel typically involves casting, followed by hot and cold rolling in a non-stop process, and culminates with continuous annealing, all of which demand significant time and energy [5,6,7,8,9].

Over the past decade, a new heat treatment method called super-fast heating has been rapidly adopted in steel production [10,11,12]. The heating rate involved in this process is dozens of times higher than the traditional heating rate, and an appropriate cooling method, such as air-jet cooling or controllable water quenching, is used to achieve the required cooling rate [13,14,15,16,17]. Super-fast heating can be achieved via several methods, such as direct-flame heating [18,19], longitudinal magnetic induction heating, and transverse magnetic induction heating [20]. Compared with traditional heat treatment processes, super-fast heating significantly reduces energy consumption [21].

As in the case of conventional heat treatment, numerous factors influence the microstructure and mechanical properties of the steel fabricated via super-fast heat treatment. These factors include heating rate, peak temperature, holding time, and cooling rate. However, these factors act through different mechanisms. Usually, super-fast heating increases the recrystallization temperature [22,23,24], resulting in grain refinement [25,26,27,28]. The peak temperature determines the phase composition and volume fraction of martensite formed during the subsequent rapid cooling, which, in turn, affects the properties of the final product [15,26,29]. The holding time affects the diffusion of carbon, which indirectly affects various processes, such as recovery, recrystallization, phase transformation, and grain growth. Numerous experimental studies have suggested a significantly shorter holding time (<1 s) when applying super-fast heat treatment to prevent abnormal grain growth [15,17,22,23]. However, during actual production, this instantaneous holding is difficult to achieve owing to limitations associated with the running speed of sheet steel [21]. To apply the experimental results to factory production, we must focus on the changes in the microstructure and properties of steel caused by super-fast heating in combination with a practically achievable holding time.

Currently, super-fast heating has been applied to various steels, such as quenched and partitioned (QP) [30,31], interstitial-free (IF) [15,28], dual-phase (DP) [32,33,34], transformation-induced plasticity (TRIP) [13,35], martensitic (Ms) [36], and press-hardening steels. Fe-0.16C-1.4Mn is a steel type in the 16MnSi system, and it can be classified as the first generation of advanced high-strength steel (AHSS). It is generally used as a TRIP-aided steel in conventional processes [37,38,39,40,41]. By setting appropriate super-fast heating process parameters, the microstructure of Fe-0.16C-1.4Mn steel can be transformed in a way similar to typical DP, TRIP, or Ms steels to meet the mechanical performance requirements of various applications. This system demonstrates broad application potential.

The strengthening mechanism of the DP structure involves not only fine-grain and second-phase strengthening but also multiple-strain hardening, including strengthening via geometrically necessary dislocations (GNDs) based on the heterostructure theory [42,43,44]. Therefore, super-fast heating and the resultant complex microstructural evolution have considerable research value.

This study aimed to investigate the effect of a fast heating rate and holding time on the microstructure and mechanical properties of Fe-0.16C-1.4Mn sheet steel. The steel was heated in the α and γ two-phase region and then rapidly cooled to obtain a microstructure similar to that of DP steel. The effects of super-fast heating on the phase transformation, recrystallization, texture, and mechanical properties were evaluated and compared with those of conventional low-speed heating. Super-fast heating parameters must be designed within a range that can be achieved in industrial production (chiefly, by including a longer holding time) so that the experimental results can be applied to industrial production.

## 2. Materials and Methods

The experimental steel used was commercial-grade Fe-0.16C-1.4Mn steel produced by Baosteel Co., Ltd. (Shanghai, China), and its chemical composition is shown in Table 1. This steel is a typical low-alloy, low-carbon steel with a low production cost and wide range of adjustable strength and ductility [45].

Figure S1 shows the phase equilibrium and cooling transformations obtained using JMatPro v7.0 for the composition in Table 1. Figure S1a shows that under equilibrium conditions, the temperature range of the two-phase region of ferrite and austenite is 695–818 °C. To ensure the formation of the desired hard phase during subsequent heat treatment, the annealing temperature should be set within this range or slightly higher to account for superheating effects. Therefore, the annealing temperature was set at 810°C. The continuous cooling transition (CCT) curve in Figure S1b shows that martensite can be formed by directly heating to 810 °C and cooling below 400 °C/s at a rate exceeding 100 °C/s. Considering the actual capacity of the continuous annealing production line, the cooling rate was set at 120 °C/s.

Annealing simulation experiments were conducted using a multifunctional annealing simulation test machine (Fives, Baosteel, Shanghai, China) under a nitrogen atmosphere. This device can simulate continuous annealing of single-piece samples. A K-type thermocouple was welded to the center of the sample to measure the temperature and precisely control the heat treatment process through electromagnetic induction heating and air-jet cooling.

The heat treatment process is divided into three stages, as shown in Figure S2. In the first stage, the test piece is heated to 300 °C at a rate of 10 °C/s and held for 30 s. This process simulates the preheating stage that removes thermal stress in sheet steel during production [21]. In the second stage, the test piece is heated to 810 °C at 5 °C/s (a conventional heating rate) or at 50, 100, or 300 °C/s (super-fast heating rate) and held for 1, 5, or 30 s to simulate sheet steel production via super-fast heating. Due to the limitation of the cooling speed of the steel plate, a minimum holding time of 5 s is required in the actual production process. The 1 s and 30 s holding times provided more options to compare the effect of different heating rates.

Mechanical property testing mainly involved tensile testing. The tensile specimens conformed to the commercial standard JIS-13A [46]. Figure S3 shows the tensile sample specification. The geometric dimension of its nominal segment is 80 mm × 25 mm × 0.5 mm. The tensile and yield strengths and elongation were tested using a universal testing machine (5581, Instron, Norwood, MA, USA) at a tensile speed of 2 mm/min with the tensile direction parallel to the cold-rolling direction.

The fracture surface after tensile testing was observed using a scanning electron microscope (EVO MA25, Zeiss, Jena, Germany). Then, metallographic specimens were prepared using standard metallographic techniques, and cross-sections parallel to the cold-rolling direction were ground and polished to a mirror finish. Their micro-Vickers hardness was tested using a micro-Vickers hardness tester (MVK-H21, Akashi, Japan) under a load of 20 g or 500 g, for a holding time of 10 s. The polished metallographic specimens were etched with a 3 vol% nitric acid–alcohol solution for 10 s, and the microstructure was observed under metallographic (Axio Imager A2m, Zeiss, Germany) and scanning electron microscopes (EVO MA25, Zeiss, Germany). The phases were characterized by electron backscatter diffraction (NordlysMax3 EBSD, Oxford Instruments, Abingdon, UK) at an acceleration voltage of 20 kV, a tilt angle of 70 °, and a step size of 0.3 μm, respectively (working distance: 16 mm and 1000× magnification). The processing of EBSD samples was the same as that of metallographic samples, except that no erosion was carried out. The elemental distributions of the etched metallographic specimens were observed via an electron probe microanalysis (EPMA 8050G, Shimadzu, Kyoto, Japan).

For phase detection, each sample was cut into thin slices with dimensions of 25 mm × 25 mm, and the phase composition and texture characteristics were analyzed using X-ray diffraction (XRD D8 Discover, Bruker, Ettlingen, Germany) after electropolishing the surface. Diffractograms were measured using a Co target, at a voltage, current, scanning speed, and step size of 30 kV, 39 mA, 2°/min, and 0.02°, respectively, from 40° to 130°. Incomplete pole figures for the {110}, {200}, and {211} reflections were measured using the reflection method on an X-ray diffractometer (Bruker, Germany, D8 Discover equipped with SC detector and Co target; 30 kV, 30 mA, φ: 0–360°, and Ψ: 20–90°). Orientation distribution function (ODF) data were calculated from the pole figure data, followed by visualization in the form of an ODF diagram.

In addition, the experimental data were processed using JMatPro v7.0 (Thermotech, Louth, UK), Channel 5 (Oxford Instrument, Abingdon, UK), and Jade 9 (Bruker, Germany) software, and the processed data were plotted using Origin 2018 (OriginLab, Northampton, MA, USA) software for further analysis and interpretation.

## 3. Results

A cold-rolled work-hardened sheet of commercial grade Fe-0.16C-1.4Mn steel was selected as the experimental substrate. Normally, cold-rolled billets are obtained from hot-rolled and annealed plates with a microstructure of massive ferrite and pearlite. After annealing, a 2 mm thick hot-rolled steel plate was cold-rolled to 0.5 mm (cold-rolled deformation: 75%). Figure S4 shows the microstructure of the cold-rolled sheet steel, which was mainly composed of deformed ferrite and deformed pearlite with an obvious fibrous texture. The cold-rolled Fe-0.16C-1.4Mn sheet steel before heat treatment had a yield strength (R_p0_._2_), a tensile strength (R_m_), an elongation (ε_f_), and a microhardness of 940 MPa, 1020 MPa, 1.5%, and 310 HV0.5, respectively.

Table 2 lists the mechanical properties of samples after different heat treatments. It indicates that heat treatment under all conditions significantly improves elongation. When the holding time was 1 or 5 s, increasing the heating rate from 5 °C/s to 300 °C/s resulted in an increase in yield and tensile strengths, with a slight decrease in elongation. When the holding time was 30 s, the change in tensile performance followed a similar pattern. However, in this case, the tensile properties of the specimens at heating rates of 100 and 300 °C/s were almost equal.

At a holding time of 1 s, the microhardness positively correlated with the heating rate. However, when the holding time was 5 or 30 s, the microhardness value peaked at a heating rate of 100 °C/s and decreased at a heating rate of 300 °C/s.

For a holding time of 30 s, at a super-fast heating rate of 300 °C/s, the yield strength, tensile strength, microhardness, and elongation of the steel were 389 MPa, 910 MPa, 298 HV0.5, and 16.5%, respectively. Compared with the attributes of the product obtained at the conventional heating rate of 5 °C/s, the yield strength, tensile strength, and microhardness increased by 55 MPa, 55 MPa, and 23 HV0.5, respectively, while only the elongation decreased by 3.1%.

**Figure 1:**
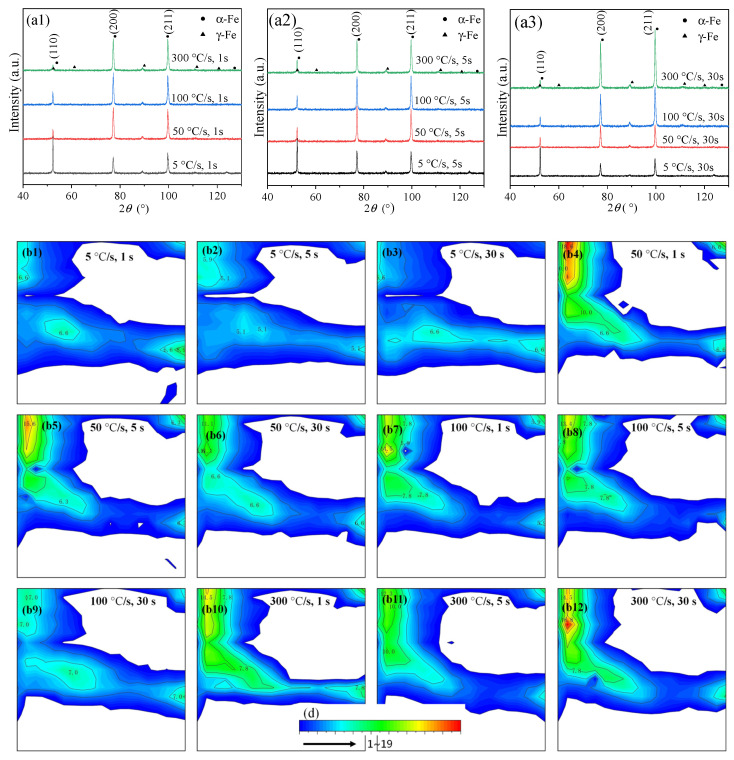

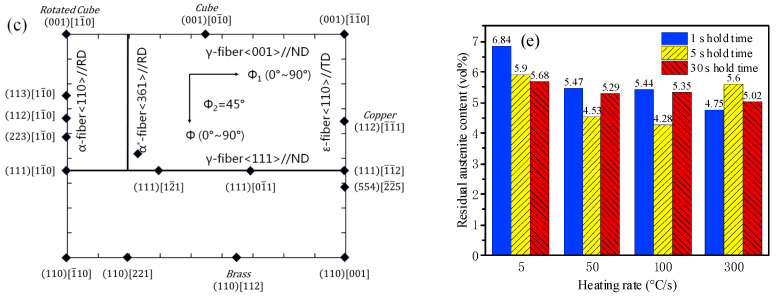
XRD patterns and ODF diagrams for φ_2_ = 45 ° of samples after heat treatments. XRD patterns: (**a1**) 5–300 °C/s for 1 s, (**a2**) 5–300 °C/s for 5 s, and (**a3**) 5–300 °C/s for 30 s; ODF diagrams: (**b1**–**b3**) 5 °C/s with holding times of 1, 5, and 30 s, respectively; (**b4**–**b6**) 50 °C/s with holding times of 1, 5, and 30 s, respectively; (**b7**–**b9**) 100 °C/s with holding times of 1, 5, and 30 s, respectively; (**b10**–**b12**) 300 °C/s with holding times of 1, 5, and 30 s, respectively; (**c**) ideal BCC texture components for φ_2_ = 45 °; (**d**) texture strength color chart; and (**e**) retained austenite fraction.

Figure 1(a1–a3) show the XRD patterns of the samples annealed at different temperatures and for different holding times, the main phases were α-Fe and γ-Fe; γ-Fe represents the residual austenite. Over any holding time, for samples heated at the conventional heating rate of 5 °C/s, the α-Fe (110) diffraction peak was the strongest, and was consistent with the diffraction characteristics of non-oriented polycrystalline body-centered cubic (BCC) materials. However, for the super-fast heating rates of 50, 100, and 300 °C/s, the diffraction peak intensities of the α-Fe (200) and (211) planes increased, while the diffraction peak intensities of the (110) plane weakened. In these cases, the materials exhibited strong anisotropy.

Figure 1(b1–b12) show the ODF diagrams of the samples subjected to different heat treatments at φ_2_ = 45° (φ2 is the angle of rotation about the sample coordinate system’s *Z*-axis (the normal direction, ND)). RD represents the cold-rolling direction, ND represents the normal direction of the cold-rolled sheet, and TD represents the transverse direction perpendicular to both the RD and ND. Comparisons of the ODF diagrams with the ideal BCC distribution diagram (Figure 1c) and intensity color bar (Figure 1d) show that the main texture components of the samples were α texture // RD and γ texture // ND [47]. Conversely, for the same holding time, the samples treated by super-fast heating at 50, 100, and 300 °C/s generally had stronger α textures than the samples treated by conventional heating at 5 °C/s, but the enhancement in the γ texture was relatively limited. At the same heating rate, a longer holding time usually weakened the α texture, but the effect on the γ texture was relatively small. Generally, α texture represents the microstructure of the rolled state, while γ texture represents the microstructure of the annealed state. A large difference between these two textures indicates that the material has a strong anisotropy [48,49]. The higher the degree of recrystallization, the weaker the α texture, and the stronger the γ texture [50,51,52]. This indicates that fast heating and a short holding time retain more of the deformed structure. The large retention of α texture with super-fast heating indicates that the degree of recrystallization was far less than that occurring under conventional slow heating.

Figure 1e shows the residual austenite content, calculated based on the XRD diffraction peaks. The residual austenite contents of the different samples were small and not significantly different, and the effect on the microstructure and properties of the material was limited.

**Figure 2:**
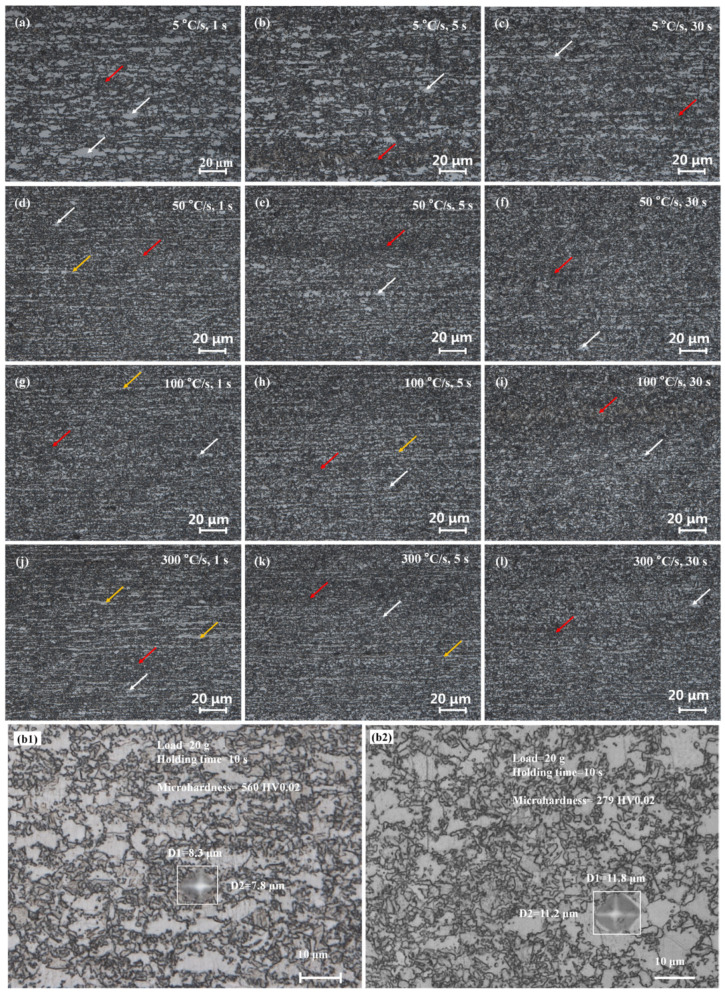
Metallographic micrographs at different heating rates (5–300 °C/s) and for different holding times (1–30 s): (**a**–**c**) 5 °C/s with holding times of 1, 5, and 30 s, respectively; (**d**–**f**) 50 °C/s with holding times of 1, 5, and 30 s, respectively; (**g**–**i**) 100 °C/s with holding times of 1, 5, and 30 s, respectively; and (**j**–**l**) 300 °C/s with holding times of 1, 5, and 30 s, respectively. Yellow, white, and red arrows indicate deformed ferrite, ferrite, and martensite, respectively. Microhardness test indentation images: (**b1**) dark and (**b2**) gray phases.

Figure 2 shows the metallographic micrographs for different heating rates and holding times. In the figure, white, red, and yellow arrows indicate ferrite, martensite, and deformed ferrite, respectively. Fe-0.16C-1.4Mn exhibited a DP morphology after super-fast heating that is consistent with the typical dual-phase steel microstructure reported in the literature [53]. Unlike the deformation microstructure shown in Figure S4, the microstructure after rapid cooling at a rate of 120 °C/s mainly consisted of ferrite and martensite.

At the conventional heating rate of 5 °C/s, numerous massive ferrite grains appeared (Figure 2a–c). In contrast, with super-fast heating at 50, 100, and 300 °C/s, not as many large ferrite domains were observed. A small amount of massive ferrite appeared in the super-fast heating samples with a holding time of 30 s (Figure 2f,i,l) and in those with a holding time and heating rate of 5 s and 50 °C/s, respectively (Figure 2e). However, in the samples subjected to super-fast heating at a holding time of 1 or 5 s, such large ferrite domains were rare (Figure 2d,g,h,j,k). Instead, there was still residual deformed ferrite. The appearance of large ferrite domains is related to recrystallization, and slow heating and a prolonged holding time contribute to such recrystallization of ferrite. This process is favorable because, with fast heating and a short holding time, austenite does not have time to nucleate and grow [54,55,56,57].

Besides ferrite, the remaining microstructure of the cold-rolled alloys consists of pearlite. When heated to 810 °C, this pearlite transforms into austenite, which again decomposes into pearlite when cooled slowly. However, at a fast cooling rate it transforms into martensite. Therefore, the dark structures shown in Figure 2 could be martensite or pearlite. Definitive identification requires the use of phase and microstructure analysis methods such as high-resolution transmission electron microscopy (HRTEM).

The nature of the dark structure in Figure 2 was indirectly confirmed by detecting the hardness of the different phases in the microstructure. The microhardness values of the different regions of the metallographic sample when subjected to a heating rate of 5 °C/s and a holding time of 5 s were measured using a microhardness tester, and the results are shown in Figure 2(b1,b2). The microhardness value of the dark area in Figure 2(b1) was as high as 560 HV0.02, which is much higher than the conventional hardness of ferrite and slightly lower than that of pure martensite [58]. This indicates that it is a composite structure of martensite and ferrite, with martensite as the main component. The microhardness value of the gray area in Figure 2(b2) was 279 HV0.02, which is very close to the conventional microhardness value of ferrite, indicating that this structure is ferrite. However, during the rapid cooling process, certain substructures may have formed in these ferrite grains, leading to an increase in their microhardness.

**Figure 3:**
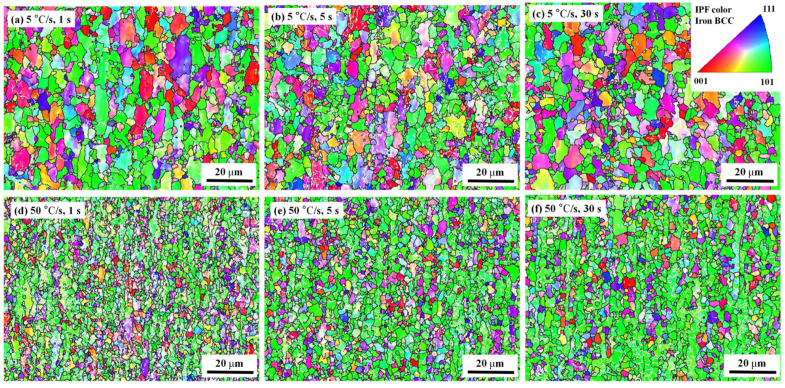

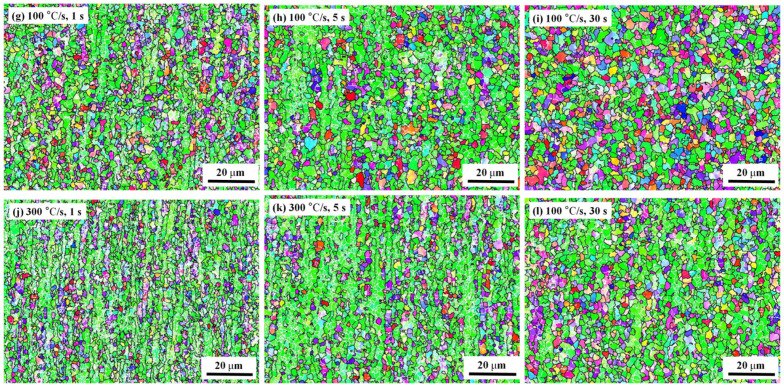
IPF maps showing the effect of heating rate (5–300 °C/s) and holding time (1–30 s) on grain boundaries: (**a**–**c**) 5 °C/s with holding times of 1, 5, and 30 s, respectively; (**d**–**f**) 50 °C/s with holding times of 1, 5, and 30 s, respectively; (**g**–**i**) 100 °C/s with holding times of 1, 5, and 30 s, respectively; and (**j**–**l**) 300 °C/s with holding times of 1, 5, and 30 s, respectively. White grain boundaries have orientation differences of 2–15° and black grain boundaries have orientation angle differences of 15–63°.

Figure 3 shows the EBSD inverse pole figure (IPF) maps of the samples after being subjected to different heat treatment conditions. Grain boundaries with small misorientation angles (MAs) are white and grain boundaries with large MAs are black. The grain colors represent the grain orientations.

At a slow-heating rate of 5 °C/s, there were few small-MA boundaries (Figure 3a–c). This indicates that many unoriented coarse grains originated from recrystallization. However, for super-fast heating, numerous small-MA boundaries were retained in band-shaped grains (white pixels in Figure 3d–l). This indicates that the large, elongated grains were formed by the merging and growth of multiple smaller grains. A comparison of the results over different holding times (Figure 3g,i) showed that when the holding time was prolonged, the small grains inside the large grains grew and began to break through the boundaries of the large grains to form new and finer equiaxed grains.

**Figure 4:**
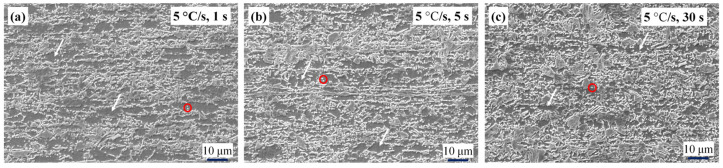

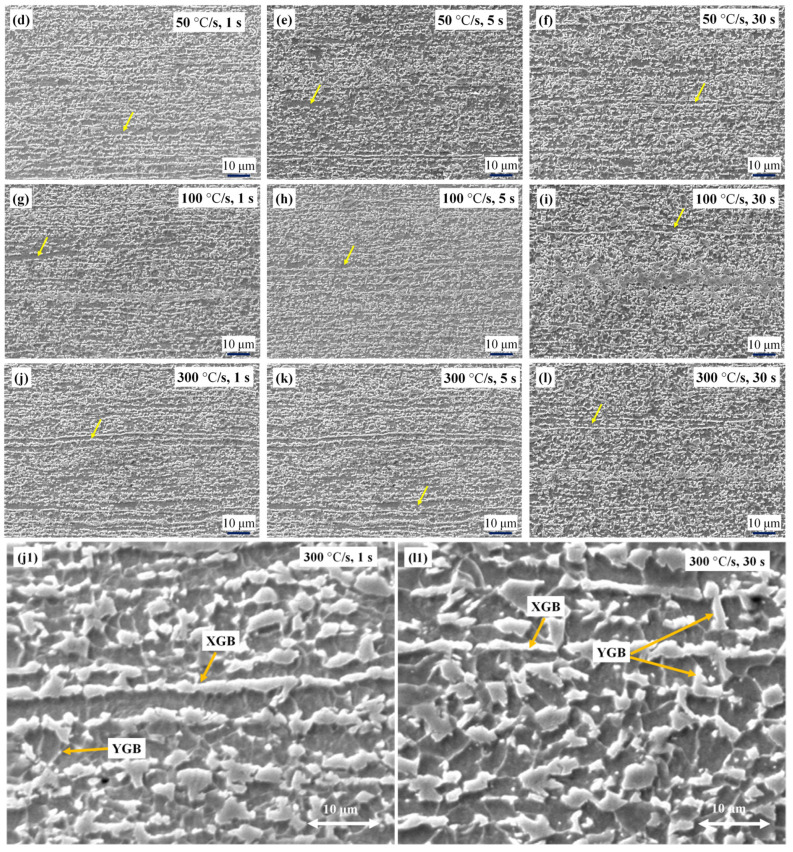
SEM images showing the effect of heating rate (5–300 °C/s) and holding time (1–30 s) on austenite transformation: (**a**–**c**) 5 °C/s with holding times of 1, 5, and 30 s, respectively; (**d**–**f**) 50 °C/s with holding times of 1, 5, and 30 s, respectively; (**g**–**i**) 100 °C/s with holding times of 1, 5, and 30 s, respectively; and (**j**–**l**) 300 °C/s with holding times of 1, 5, and 30 s, respectively. The yellow, white, and red circles in these images represent deformed ferrite (DF), recrystallized ferrite (RF), and spheroidized cementite, respectively. Images (**j1**) and (**l1**) show the axial grain growth characteristics of samples heated at 300 °C/s for 1 s and 30 s, respectively.

Figure 4 shows the scanning electron microscopy (SEM) images of the microstructures of the samples after different heat treatments, with the dark regions representing ferrite grains, while the bright regions represent the martensite structures formed from supercooled austenite. For the samples heated at 5 °C/s (Figure 4a–c), the ferrite grains (white arrows) remained equiaxed regardless of the holding time, indicating the completion of recrystallization before the phase transformation.

For the samples treated at heating rates of 50 and 100 °C/s (Figure 4d–i), some banded grains (yellow arrows) were still retained, indicating that the ferrite only partially recrystallized before the martensitic transformation. Furthermore, for the samples treated at a heating rate of 300 °C/s (Figure 4j–l), fibrous grains with more pronounced sizes were clearly observed along the rolling direction, indicating a significant increase in the recrystallization temperature. For the same holding time, rapid heating reduced the degree of recrystallization of the sheet steel, which is consistent with the results of the texture analysis shown in Figure 1.

## 4. Discussion

### 4.1. Phase Transformation and Growth of Austenite

In this study, the cooling rate of 120 °C/s is close to the general water quenching rate, which is approximately 150 °C/s. Therefore, austenite is most likely to transform into martensite. This can also explain the significantly low residual austenite content, as shown in Figure 1e. The phase transformation and growth of austenite during continuous super-fast heating can be studied by observing the morphology, distribution, and content of martensite and ferrite at room temperature.

Austenite tends to nucleate at the ferrite phase boundaries, usually because the strain energy is higher at the boundary. Nucleation can also eliminate crystal defects and reduce the free energy. Furthermore, the strain energy generated during the nucleation of new austenite grains can be more readily eliminated through phase-transition rheology. For the samples heated at the conventional rate of 5 °C/s (Figure 4a–c), the cementite lamellae in the original pearlite and cementite particles distributed at the ferrite grain boundaries were transferred to ferrite grains and spheroidized (red circles). Because austenite tends to nucleate at the original ferrite grain boundary, the resultant martensite clusters were mainly distributed along the ferrite grain boundaries [59]. When the holding time was extended, austenite had sufficient time to grow; therefore, the martensite clusters had larger dimensions and occupied a greater volume fraction, resulting in the more finely fragmented ferrite grains shown in Figure 4c when compared with Figure 4a.

As shown in Figure 4d–i, the microstructures of the samples treated at 50, 100, and 300 °C/s were similar. At relatively high heating rates, austenite mainly nucleated at the two-phase interface between ferrite and cementite [60]. Additionally, partially nucleated austenite migrated to the interior of ferrite during ferrite growth, fragmenting the original ferrite structure into finer domains.

Conversely, for the steel treated at 50, 100, and 300 °C/s, the growth rates of austenite along the axial ferrite grain boundaries (XGBs) were significantly faster. Figure 4(j1,l1) show the axial growth characteristics of the grains during super-fast heating. When the XGBs were fully occupied, some longitudinal ferrite grain boundaries (YGBs) remained, as shown in Figure 4(j1), indicating that the diffusion rate of atoms along different grain boundaries differed. As per Figure 3d–l, XGBs are the grain boundaries with orientation angle differences of 15–63°; the atoms preferentially diffused along the grain boundaries with larger orientation angle differences. This is probably because the structural disorder of such grain boundaries was greater, and carbon atoms diffused more rapidly. Overall, austenite preferentially grew along the grain boundaries oriented in the rolling direction.

As shown in Figure 4j–l, after the super-fast heating at 300 °C/s, the difficulty in the completion of the recrystallization process resulted in a large number of cementite particles being distributed at the grain boundaries, which provided favorable positions for austenite nucleation. The austenite formed along the rolling direction hindered the longitudinal growth of ferrite grains, eventually forming fiber-like ferrite grains. Extending the holding time can provide sufficient time for austenite to grow along the YGBs to a certain extent (Figure 4(l1)), thereby eliminating the fiber-like grain structure.

**Figure 5:**
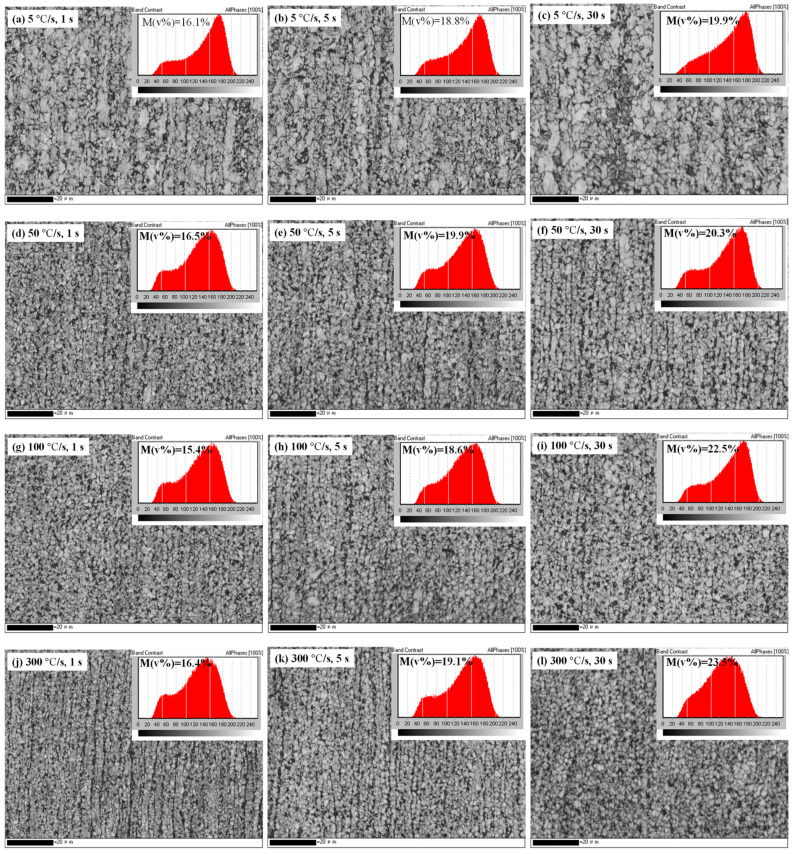
EBSD band contrast images of samples after different heat treatment processes: (**a**–**c**) 5 °C/s with holding times of 1, 5, and 30 s, respectively; (**d**–**f**) 50 °C/s with holding times of 1, 5, and 30 s, respectively; (**g**–**i**) 100 °C/s with holding times of 1, 5, and 30 s, respectively; and (**j**–**l**) 300 °C/s with holding times of 1, 5, and 30 s, respectively. The inset in each picture shows the distribution of the band contrast for all diffraction peaks.

The volume fraction of martensite reflects the different nucleation and growth modes of austenite. Figure 5 shows the band contrast (BC) images of the samples subjected to different heat treatments. In the BC images, the lighter areas indicate grains with a perfect crystal lattice, whereas the darker areas indicate defects and strain.

The inset in each image in Figure 5 shows the distribution of the band contrast for all diffraction peaks. The diffraction patterns are superpositions of martensite and ferrite peaks. The martensite substructure is richer than that of ferrite, so the volume fraction of martensite in each sample can be estimated by separating the diffraction peaks using an integral method. The statistical results are shown in Table 3. It is shown that under all heating rates, the martensite content significantly increased with a longer holding time. When the holding time was extended from 1 to 5 s, the martensite content increased rapidly, but when it was extended from 5 to 30 s, the increase in martensite content slowed. Increasing the heating rate increased the martensite content, with the highest content occurring at a heating rate of 300 °C/s and for a holding time of 30 s. Martensite content is closely related to austenite growth, and the phase transformations and growth of austenite essentially involve the diffusion of carbon atoms. Therefore, understanding the increase in martensite content requires further investigation of carbon diffusion [54,61,62].

**Figure 6:**
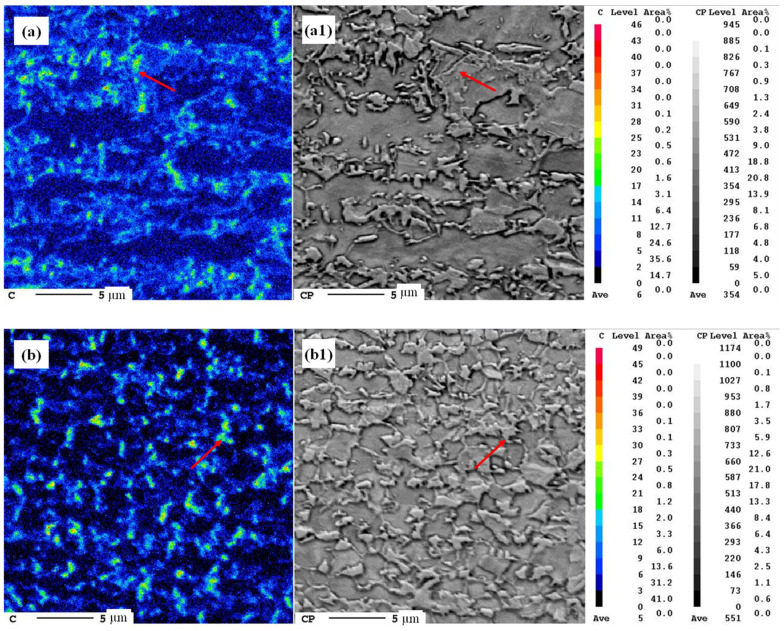
Carbon distribution measured by EPMA for samples treated at different heating rates: EPMA image of (**a**) 5 °C/s, 1 s and (**b**) 300 °C/s, 1 s; red arrows indicate the high carbon areas. Images (**a1**,**b1**) are SEM of the corresponding areas. C level is the color level used to identify the clustering characteristics of C in EMPA images, while CP level is interpreted as the color profundity level, which is the color depth of SEM and reflects the shape characteristics of the phases.

Figure 6 shows the carbon distribution obtained by EPMA surface scanning (red arrows indicate high-carbon areas). An analysis of the distribution of carbon in the microstructure of the samples heated at different rates is helpful for further studying the diffusion behavior of carbon atoms during the austenite phase transformation and growth processes.

Figure 6(a,a1) show that when the 5 °C/s heating process was used, the high-carbon area was generally distributed at the phase interface between ferrite and martensite, indicating that the growth of austenite during slow heating mainly relied on the carbon atoms from the cementite at the original ferrite grain boundary; carbon diffused from the ferrite grain boundary to the austenite. As shown in Figure 6(b,b1), compared with the slow-heating samples, the samples subjected to super-fast heating had stronger carbon clustering, but the original austenite had a higher carbon concentration. This is probably because, with super-fast heating, ferrite does not have time to complete recrystallization, resulting in the formation of a large number of cementite domains during rolling. Therefore, when austenite grew, it grew into the interior of ferrite, and the distribution of carbon in austenite was initially uneven. During austenite growth, carbon atoms diffused from the original cementite region to the original ferrite region. In other words, to homogenize the carbon concentration and reach a relatively high concentration in the center of the austenite, carbon atoms must diffuse from what was initially the phase interface.

The different austenite growth mechanisms lead to changes in the martensite content, as shown in Table 3. Here, the evolution is divided into three stages.

Stage 1: In the initial stage of phase transformation, i.e., at the beginning of the holding period, the martensite contents in the samples treated at 5 and 50 °C/s are even higher than those in the samples treated at 100 and 300 °C/s. This is because the growth rate of austenite along the ferrite grain boundaries is significantly high, and a slower heating rate is more likely to reach equilibrium. Notably, the process design shown in Figure S2 allows the materials to remain in a given temperature range for a longer period with a low heating rate. Therefore, in the initial stage of phase transformation, the austenite content of the materials treated at 5 and 50 °C/s was higher.

Stage 2: When the holding time is short, with a heating rate of 5 °C/s, carbon atoms around the austenite nuclei aggregate, resulting in a significant carbon concentration difference at the phase interface; however, with a heating rate of 300 °C/s, a large number of austenite nuclei form in a short time and there is a high concentration difference inside, so the martensite content increases rapidly with holding time for the samples heated at both 5 and 300 °C/s.

Stage 3: When the holding time is greater than 5 s, the growth rate of austenite slows due to the decrease in the carbon concentration difference. However, at this stage, the martensite content varies significantly with different heating rates. The martensite content of the materials treated at 100 and 300 °C/s is much higher than that of the materials treated with slow heating. There are two reasons for this. First, the overheating resulting from rapid heating promotes the nucleation of a large amount of austenite and reduces the average diffusion distance of carbon atoms; second, the degree of recrystallization is very small in the case of rapid heating, resulting in the retention of more deformation substructures that provide channels for the diffusion of carbon atoms.

### 4.2. Mechanical Properties

Table 2 shows the relationship between tensile mechanical properties, microhardness, heating rate, and holding time. For the same holding time, the yield and tensile strengths increased with an increase in the heating rate, whereas the elongation at break decreased. However, the steel with a holding time of 1 s had higher strength and lower elongation than the steel with longer holding time.

For the steel with a holding time of 1 s, when the heating rate was increased from 5 to 50 °C/s, the yield and tensile strengths increased rapidly, while the elongation decreased rapidly. In contrast, when the heating rate was increased from 50 to 100 °C/s, there was little change in the tensile properties. When the heating rate was further increased to 300 °C/s, the yield and tensile strengths significantly increased further, while the elongation decreased significantly.

For the steels with holding times of 5 or 30 s, when the heating rate was increased from 5 to 50 °C/s, the yield and tensile strengths increased rapidly, while the elongation decreased rapidly. Then, when the heating rate was increased from 50 to 300 °C/s, the tensile properties remained almost unchanged.

The microhardness trends were significantly different from those of the tensile properties. In particular, for the steel with a holding time of 1 s, the microhardness slightly increased with an increase in the heating rate, but for the steel with holding times of 5 and 30 s, the highest microhardness values were obtained when heated at 100 °C/s. The steel with a holding time of 30 s showed higher microhardness when heated at 50 or 100 °C/s, and the microhardness rapidly decreased when heated at 300 °C/s.

**Figure 7:**
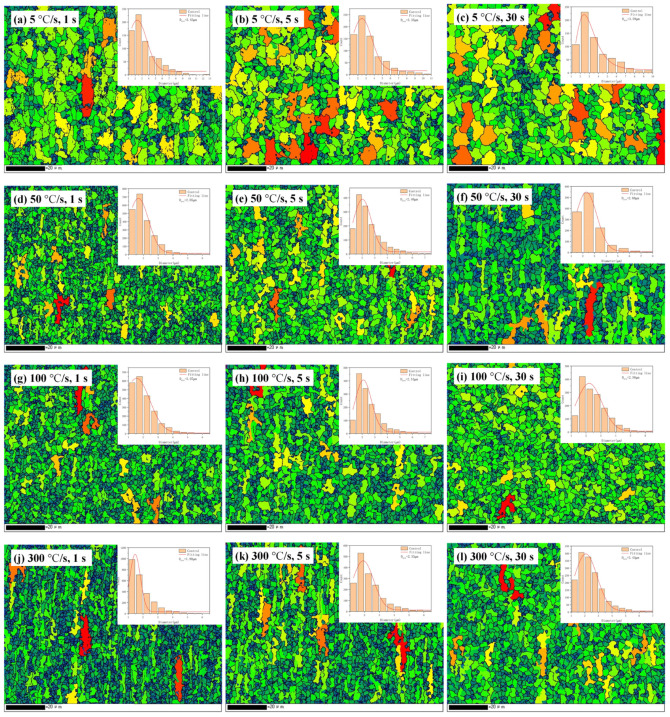
Frequency distribution of grain size of samples after different heat treatments: (**a**–**c**) 5 °C/s with holding times of 1, 5, and 30 s, respectively; (**d**–**f**) 50 °C/s with holding times of 1, 5, and 30 s, respectively; (**g**–**i**) 100 °C/s with holding times of 1, 5, and 30 s, respectively; and (**j**–**l**) 300 °C/s with holding times of 1, 5, and 30 s, respectively. The smaller the grains, the greener the color; the larger the grains, the redder the color.

The grain size has a significant impact on the mechanical properties of materials. Figure 7 shows the grain size distributions of the samples subjected to different heat treatment processes (grain size refers to the equivalent-circle diameter of the grain). It is shown that an increase in the heating rate significantly refined the grain size and retained more fibrous grains. The weighted average grain sizes were calculated from the distributions plotted in Figure 7, and the results are summarized in Table 4. Across all holding times, the relationship between the grain size and heating rate exhibited a similar trend. When the heating rate was increased from 5 to 50 °C/s, the average grain size significantly decreased; when the heating rate was increased from 50 to 300 °C/s, the average grain size barely changed.

For a heating rate of 5 °C/s, the effect of holding time on grain size was insignificant. In contrast, for heating rates of 50, 100, and 300 °C/s, increased holding times resulted in increased grain sizes, probably because a heating rate of 5 °C/s means that the steel has been heated for a longer period of time; so, a short increase in holding time will not have a significant impact. However, during super-fast heating, there is insufficient time for the recrystallization of ferrite grains and growth of austenite grains. Therefore, appropriately increasing the holding time provides opportunities for the growth of these grains.

According to the Hall–Petch equation (Equation (1)) [63],

(1) $$\sigma_{s}=\sigma_{0}+\frac{k_{y}}{\sqrt{d}},$$

where $\sigma_{s}$ represents the yield strength, $\sigma_{0}$ represents the lattice friction resistance of a single dislocation, $k_{y}$ represents a constant related to the material composition, and d represents the average grain diameter. The smaller the average grain size, the higher the yield strength. When the heating rate was increased from 5 to 50 °C/s, the strength of the samples shown in Table 2 significantly increased due to a significant decrease in grain size.

The dislocation density within grains has a significant impact on the microhardness value. The full width at half maximum (FWHM) of the diffraction peaks in the XRD pattern is used to characterize the dislocation density. The relationship between the FWHM and dislocation density can be expressed by a modified Williamson–Hall equation [64,65,66,67,68], as shown in Equations (2) and (3).

(2) $$∆K_{tot}=\frac{0.9}{L}+{\left(\frac{\pi A^{2}b^{2}}{2}\right)}^{\frac{1}{2}}\rho^{\frac{1}{2}}\left(K{\bar{C}}^{\frac{1}{2}}\right),$$

(3) $$\bar{C}={\bar{C}}_{h00}\left(1-q\frac{h^{2}k^{2}+k^{2}l^{2}+h^{2}l^{2}}{h^{2}+k^{2}+l^{2}}\right).$$

Here, $∆K_{tot}$ represents the FWHM; L represents the coherent domain size; A represents the constant related to the effective outer cut-off radius of the dislocation; b represents the Burgers vector; ρ represents the dislocation density; K represents the peak position; $\bar{C}$ represents the average contrast factor of dislocations on a specific crystal plane; h, k, and l are Miller factors of diffraction crystal planes; and ${\bar{C}}_{h00}$ and q are constants that depend on the lattice elastic constant.

As per Equations (2) and (3), there is a positive correlation between the FWHM and dislocation density. Based on the XRD patterns in Figure 1, the FWHM at 2*θ* = 52.377 ° was calculated for the steel with different heat treatment processes, and the values are shown in Table 5.

For the samples with a holding time of 1 s, the FWHM increased slightly when the heating rate was increased from 5 to 100 °C/s and rapidly when the heating rate was increased from 100 to 300 °C/s. This indicates that for samples with a short holding time, a slow heating process plays an important role in the recovery of substructures, such as dislocations within the grains. In contrast, super-fast heating at 300 °C/s does not provide sufficient recovery time; hence, a high density of substructures is retained. This contributes to a high microhardness and strength, but it is not conducive to the improvement of elongation.

**Figure 8:**
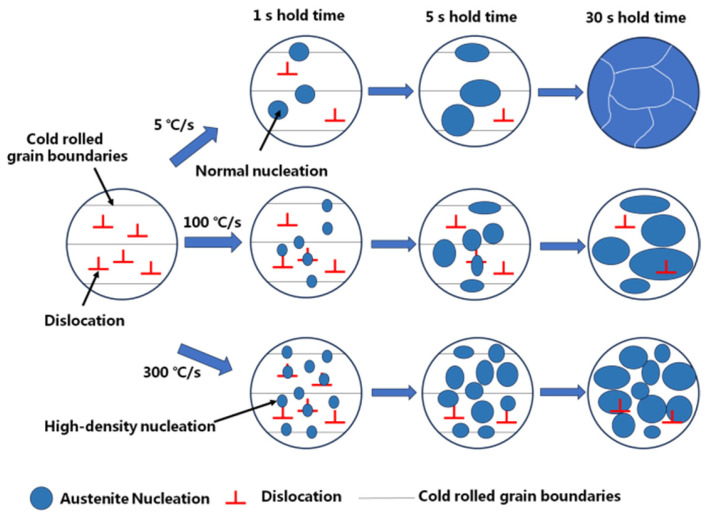
Schematic diagram of dislocation recovery and austenite evolution during different heat treatment processes.

For samples with holding times of 5 and 30 s, the highest FWHM was obtained with the 100 °C/s heating process. When the heating rate was increased from 5 to 100 °C/s, the FWHM increased because the increase in heating rate caused more substructures to remain in the two-phase region. However, the FWHM decreased for a heating rate of 300 °C/s. This is because super-fast heating led to a large number of austenite nuclei at the original defect site, and the longer holding time allowed sufficient growth of austenite to eliminate these dislocations. Thus, the dislocation densities of these samples were even lower than those of the samples heated at 100 °C/s. Figure 8 shows a schematic of the dislocation recovery and austenite evolution during different heat treatments.

When heated at 100 °C/s, the microhardness of steel with a holding time of 30 s was much higher than that of steel with a holding time of 5 s, probably due to the formation of a larger amount of austenite during the longer holding process, ultimately leading to a larger amount of martensite, as shown in Table 3. When the heating rate was increased from 100 to 300 °C/s, the combined effect of the changes in dislocation density and grain-size strengthening resulted in only a minor change in tensile properties and significant decrease in microhardness.

**Figure 9:**
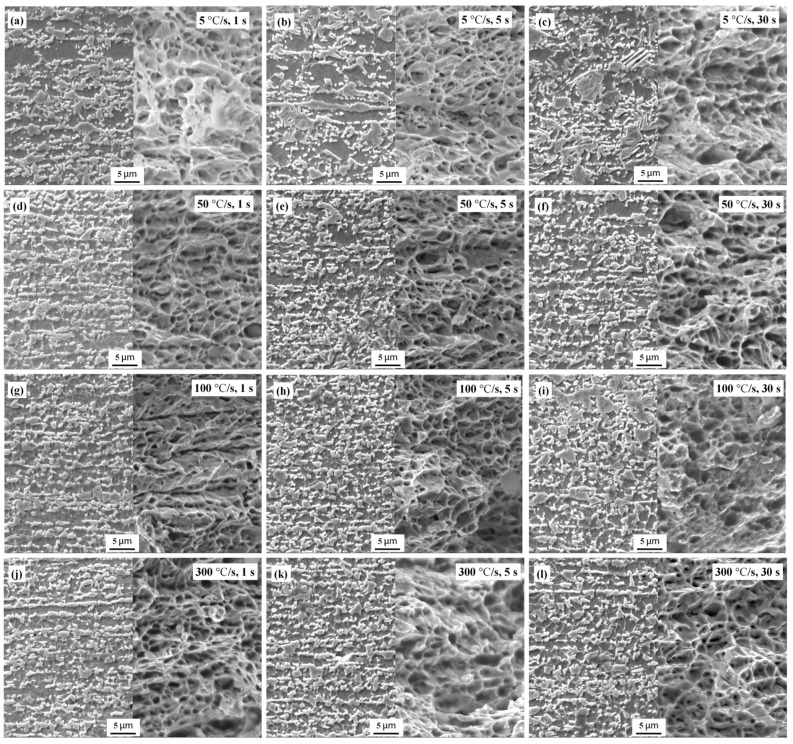
Tensile fracture morphology and microstructure of the material showing the influence of the heat treatment process on the fracture behavior: (**a**–**c**) 5 °C/s with holding times of 1, 5, and 30 s, respectively; (**d**–**f**) 50 °C/s with holding times of 1, 5, and 30 s, respectively; (**g**–**i**) 100 °C/s with holding times of 1, 5, and 30 s, respectively; and (**j**–**l**) 300 °C/s with holding times of 1, 5, and 30 s, respectively. The left side of each figure shows the microstructure, and the right side shows the corresponding tensile fracture morphology.

Figure 9 shows SEM comparisons of the microstructure and fracture morphology of the steels for each heat treatment process. The left side of each image shows the metallographic structure, and the right side shows the corresponding fracture morphology.

Figure 9a–c clearly show that a large number of plastic deformation regions appeared in the fracture morphology of the samples heated at 5 °C/s. As shown in Figure 9d–l, the samples heated at 50, 100, and 300 °C/s exhibited more brittle fracture characteristics compared to the samples heated at 5 °C/s. The ductile dimples on the fracture surface were slightly larger than the recrystallized ferrite domains, possibly because of the plastic strain on the recrystallized ferrite during the tensile process. Sharp tear ridges around the dimples show the martensite regions adjacent to the ferrite regions, which are consistent with ferrite withstanding higher strain and lower stress, and with martensite withstanding higher stress and lower strain. This indicates the presence of a certain strain distribution and stress gradient between different phases when fracture occurs, which has a profound impact on the elongation. At the microstructure level, the coarse-grained ferrite encapsulated by the fine-grained martensite resembles an atypical harmonic heterostructure [69,70]. In harmonic heterostructures, cracks are more likely to initiate at fine-grained interfaces because of the large grain boundary area in the fine-grained regions and typically lower plastic deformation capability of fine grains [71]. Subsequently, cracks tend to propagate along the fine-grained regions because of the relatively low cohesive strength of the fine grain boundaries [72].

**Figure 10:**
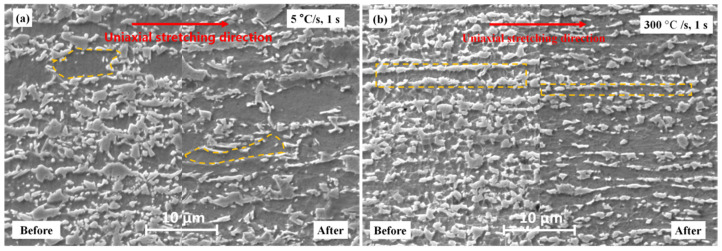
Microstructures of the samples before and after tensile tests showing the plastic deformation of the grains: (**a**) 5 °C/s, 1s and (**b**) 300 °C/s, 1s. The uniaxial stretching direction is marked with a red arrow. The left side of each picture shows the morphology before stretching, and the right side shows the morphology after stretching. The dark phase is recrystallized ferrite, and the light phase is martensite.

Figure 10 shows the microstructure of the specimen before and after the tensile test to illustrate the strain distribution between different phases during the deformation process. In each figure, the left and right sides show the grain morphologies before and after tensile testing, respectively. In these figures, the morphology of some ferrite grains is described by orange dotted lines. As shown in Figure 10a, after uniaxial tensile test, the main feature of the 5 °C/s sample was the significantly increased aspect ratio of slender ferrite grains, indicating that the softer ferrite underwent severe plastic deformation. Conversely, the hard martensite region flowed only along the phase interface, and the morphology of individual martensite grains remained unchanged, indicating that they mainly deformed elastically, potentially with a small amount of plastic deformation.

As shown in Figure 10b, after uniaxial tensile testing, the typical fibrous grains in the 300 °C/s sample became more elongated, the distance between the longitudinal ferrite grain boundaries increased, and the martensite clusters at the grain boundaries were dispersed. Because there was limited equiaxed recrystallized ferrite in this super-fast heating sample, the plastic deformation of ferrite was not as significant as that of the samples heated at 5 °C/s. The different deformation modes of ferrite and martensite during the tensile test led to differences in the strain distribution between the two phases [73]. Because strain must be continuous at the interface [74], this strain distribution generates entangled dislocations within the grains, suppressing macroscopic necking of the material [42,75]. This microstructure not only deflects cracks during deformation, but also increases strain hardening, which can effectively improve elongation [76,77]. This toughening mechanism is similar to that of non-uniform lamellar heterogeneous structures [73]. Thus, the samples treated by the 5 °C/s heating process had higher ductility and elongation compared to those of the samples treated by the 100 °C/s and 300 °C/s heating processes.

Current research has clarified the impact of rapid heating on the microstructure and properties of Fe-1.6C-1.4Mn steel. In the future, the heterogeneous microstructure of Fe-1.6C-1.4Mn steel with partial recrystallization of the laminated structure will be prepared through rapid heating processes, combined with low-temperature annealing and controlled water quenching technology, to further improve its mechanical properties.

## 5. Conclusions

1. The microstructure of the DP Fe-0.16C-1.4Mn steel subjected to rapid heating and cooling is primarily composed of ferrite and martensite, along with a small amount of residual austenite. The heating rate affects the degree of recrystallization and nucleation mode of austenite. For slow heating (5 °C/s), the degree of recrystallization is high when entering the two-phase region, and austenite nucleates at the grain boundaries of recrystallized ferrite. For the super-fast heating rates (50, 100, and 300 °C/s), little recrystallization occurs before entering the two-phase region, and austenite nucleates on the cementite particles at the original fibrous ferrite grain boundaries.
2. The morphology of martensite is related to the growth of austenite during heating. In the fast heat treatment processes, the heating rate can affect the growth of austenite in the two-phase region of the dual-phase Fe-0.16C-1.4Mn steel. At a heating rate of 5 °C/s, carbon atoms diffuse from carbides to the austenite nucleation region and concentrate at the austenite boundaries, resulting in smaller martensite domains. At a heating rate of 300 °C/s, carbon atoms diffuse from the original cementite region to the ferrite region within the austenite and concentrate in the center of austenite, resulting in smaller martensite domains.
3. Compared to heating at 5 or 50 °C/s, the holding time has a greater impact on the martensite content when heating at 100 or 300 °C/s. The 100 °C/s and 300 °C/s processes retain more substructures, facilitating the nucleation of a large amount of austenite in the two-phase region, and many austenite grains rapidly grow during the subsequent holding period, resulting in a significant increase in the martensite volume fraction with increasing holding time.
4. In this study, the Fe-0.16C-1.4Mn DP steel achieved the most balanced mechanical properties at a heating rate 100 °C/s for a holding time of 5–30 s. The yield and tensile strengths were ~390 and 900 MPa, respectively. Furthermore, the elongation and Vickers microhardness were over 17% and 280 HV0.5, respectively. With an increase in the heating rate, the yield strength, tensile strength, and elongation decreased. When the holding time was extremely short (1 s), the 300 °C/s process retained a large amount of cold-rolling substructure, resulting in a high strength and hardness but low elongation. For longer holding times (5 or 30 s), compared with heating at 100 °C/s, austenite nucleated in larger quantities in the substructure during heating at 300 °C/s, and this reduced the dislocation density of the material during subsequent growth, resulting in a decrease in Vickers microhardness.
5. The Fe-0.16C-1.4Mn DP steel heated at 5 °C/s exhibited more strain partitioning during uniaxial tensile testing compared to that heated at 300 °C/s. Large recrystallized ferrite grains underwent more plastic deformation than martensite clusters, which, to some extent, suppressed necking and led to better elongation.

## Figures and Tables

**Table 1:** Chemical composition of experimental Fe-0.16C-1.4Mn sheet steel (wt.%).

C	Si	Mn	P	S	Alt	N	Nb	Ti	Fe
0.16	0.2	1.4	<0.01	<0.01	0.033	<0.01	0.01	0.015	Bal.

**Table 2:** Mechanical properties of samples after different heat treatments.

Heating Rate (°C/s)	Held Time (s)	R_p0_._2_ (MPa)	Confidence Intervals R_p0_._2_	R_m_ (MPa)	Confidence Intervals R_m_	ε_f_ (%)	Confidence Intervals ε_f_	Micro Hardness (HV0.5)	Confidence Intervals HV
5	1	356	2	866	2	19.0	0.5	276	4
50	1	398	13	907	5	16.6	0.6	275	3
100	1	395	2	909	1	16.9	1.4	277	1
300	1	467	4	935	1	11.1	0.7	286	5
5	5	364	13	865	8	19.8	1.1	261	4
50	5	381	3	896	2	18.0	0.7	277	9
100	5	382	1	898	4	17.8	0.6	283	3
300	5	387	1	913	3	17.5	1.0	276	5
5	30	334	11	865	5	19.6	0.6	275	1
50	30	379	12	898	10	17.2	0.9	282	3
100	30	389	1	910	4	16.5	1.8	298	5
300	30	389	9	907	11	16.3	1.7	277	2

**Table 3:** Martensite content estimated by the integral method (vol.%).

Held Time (s)	Heating Rate (°C/s)			
	5	50	100	300
1	16.1	16.5	15.4	16.4
5	18.8	19.9	18.6	19.1
30	19.9	20.3	22.5	23.5

**Table 4:** Average grain sizes of samples treated with different heat treatment processes, (μm).

Held Time (s)	Heating Rate (°C/s)			
	5	50	100	300
1	3.43	2.05	2.07	1.90
5	3.55	2.48	2.51	2.33
30	3.59	2.68	2.50	2.43

**Table 5:** Diffraction peak widths (FWHM at 2*θ* = 52.377 °) of samples after heat treatments (°).

Held Time (s)	Heating Rate (°C/s)			
	5	50	100	300
1	0.407	0.417	0.42	0.473
5	0.41	0.427	0.443	0.425
30	0.401	0.431	0.447	0.425

## Data Availability

The original contributions presented in the study are included in the article and Supplementary Materials, further inquiries can be directed to the corresponding authors.

## Supplementary Materials

The following supporting information can be downloaded at: https://www.mdpi.com/article/10.3390/ma17204982/s1, Figure S1: (a) Equilibrium phase diagram and (b) CCT diagram of Fe-0.16C-1.4Mn calculated using JMatProv; Figure S2: Schematic representation of the different heat treatments applied to the Fe-0.16C-1.4Mn steel; Figure S3: JIS-13A mechanical tensile sample specification; Figure S4: Initial ferrite and pearlite structure of Fe-0.16C-1.4Mn after 75% cold rolling, showing gray ferrite and black pearlite clusters.

## Abbreviations

ODF	Orientation distribution function
XRD	X-ray diffraction
EPMA	Electron probe microanalysis
EBSD	Electron backscatter diffraction
CCT	Continuous cooling transition
GND	Geometrically necessary dislocation
QP	Quenched and partitioned
IF	Interstitial-free
DP	Dual-phase
TRIP	Transformation-induced plasticity
Ms	Martensitic
AHSS	Advanced high-strength steel
BCC	Body-centered cubic
HRTEM	High-resolution transmission electron microscopy
IPF	Inverse pole figure
MA	Misorientation angle
SEM	Scanning electron microscopy
XGB	Axial ferrite grain boundary
YGB	Longitudinal ferrite grain boundary
BC	Band contrast
FWHM	Full width at half maximum
