# Supplementary material for: The Effects of Super-Fast Heating Rate and Holding Time on the Microstructure and Properties of DP Fe-0.16C-1.4Mn Sheet Steel
Source: Materials (Basel). 2024 Oct 11;17(20):4982. doi: 10.3390/ma17204982 (PMC11509179; doi:10.3390/ma17204982)
Supplement: Supplementary file 1 [file materials-17-04982-s001.zip › materials-3235365-supplementary.pdf]

The Effects of Super-Fast Heating Rate and Holding Time on the Microstructure and Properties of DP Fe-0.16C-1.4Mn Sheet Steel

Jiazheng Zhao [1], Jian Wang [2], Jun Li [2], Shengen Zhang [1,*] and Fenghua Luo [3,*]

1 Institute for Advanced Materials and Technology, University of Science and Technology Beijing, Beijing 100083, China

2 Central Research Institute of Baosteel Group, Shanghai 201900, China

3 State Key Laboratory of Powder Metallurgy, Central South University, Changsha 410083, China

* Correspondence: zhangshengen@mater.ustb.edu.cn (S.Z.); fenghualuo@csu.edu.cn (F.L.)

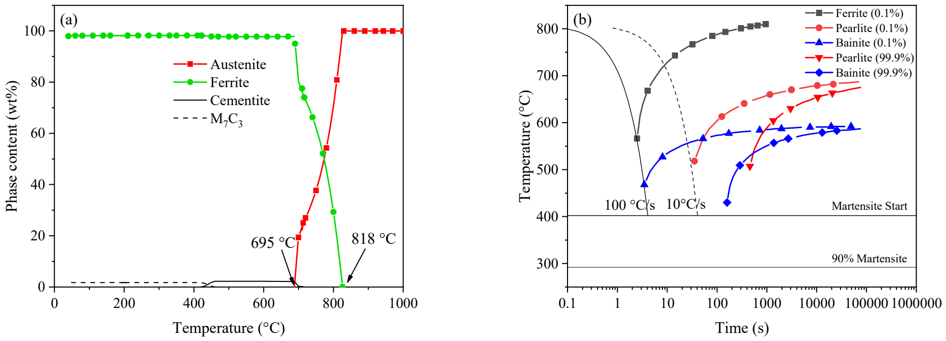

Figure S1. (a) Equilibrium phase diagram and (b) CCT diagram of Fe-0.16C-1.4Mn calculated using JMatProv 7.0.

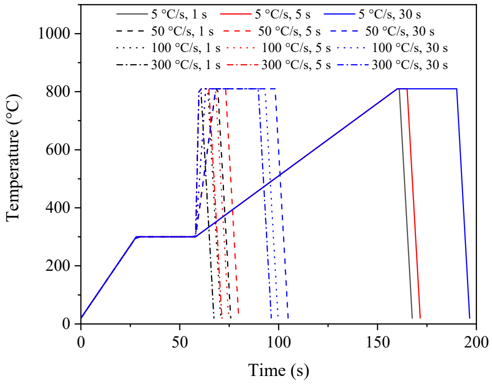

Figure S2. Schematic representation of the different heat treatments applied to the Fe-0.16C-1.4Mn steel.

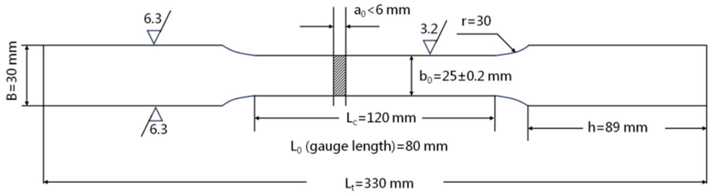

Figure S3. JIS-13A mechanical tensile sample specification

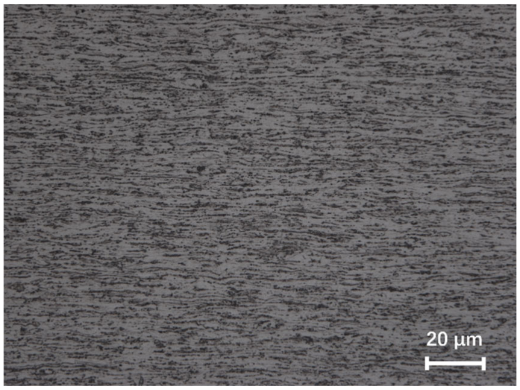

Figure S4. Initial ferrite and pearlite structure of Fe-0.16C-1.4Mn after 75% cold rolling, showing gray ferrite and black pearlite clusters.
